# Supplementary material for: Programmable spatial deformation by controllable off-center freestanding 4D printing of continuous fiber reinforced liquid crystal elastomer composites
Source: Nat Commun. 2023 Jun 30;14:3869. doi: 10.1038/s41467-023-39566-3 (PMC10313695; doi:10.1038/s41467-023-39566-3)
Supplement: Supplementary file 2 — Supplementary Information [file 41467_2023_39566_MOESM2_ESM.pdf]

Supplementary Information

Programmable Spatial Deformation by Controllable Off-center Freestanding 4D Printing of Continuous Fiber Reinforced Liquid Crystal Elastomer Composites

Qingrui Wang, Xiaoyong Tian*, Daokang Zhang, Yanli Zhou, Wanquan Yan, and Dichen Li

State Key Laboratory for Manufacturing Systems Engineering, Xi'an Jiaotong University, Xi'an, Shaanxi, 710049, China

* Corresponding author. Email: leoxyt@mail.xjtu.edu.cn

This PDF file includes:

Supplementary Discussion

Supplementary Figures 1 to 7

Supplementary Table 1

Supplementary Discussion: Calculation of composites bending curvature.

When the lateral force on the fiber bundle is almost zero, the cross-section of the fiber bundle can be approximately circular and off-center distributed on one side of the liquid crystal filament. Let the cross-sectional radius of the fiber bundle and the liquid crystal be *r* and *R*, respectively. The distance between the neutral surface of bending deformation and the central axis of the filament is set as *h*. Calculate the force balance equation of the composite:

$$\begin{aligned} & E_R\int_0^R\int_0^{2\pi}[\rho\sin\theta - h - \frac{1}{k}(1+\alpha_R\Delta T)]\rho \mathrm{d}\theta \mathrm{d}\rho \\ & + E_r\int_0^r\int_0^{2\pi}[\rho\sin\theta - h + R - r - \frac{1}{k}(1+\alpha_r\Delta T)]\rho \mathrm{d}\theta \mathrm{d}\rho = 0 \end{aligned} \tag{1}$$

where ρ and θ are two polar coordinate system parameters, and α_R and α_r represent the CTEs of LCE and fiber respectively. And then calculate the moment equation of the composite:

$$\begin{aligned} & E_R\int_0^R\int_0^{2\pi}[\rho\sin\theta - h - \frac{1}{k}(1+\alpha_R\Delta T)]\rho^2 \sin\theta \mathrm{d}\theta \mathrm{d}\rho \\ & + E_r\int_0^r\int_0^{2\pi}[\rho\sin\theta - h + R - r - \frac{1}{k}(1+\alpha_r\Delta T)](\rho\sin\theta + R - r)\rho \mathrm{d}\theta \mathrm{d}\rho = 0 \end{aligned} \tag{2}$$

Using the above two equations to eliminate *h*, and the equation can be obtained:

$$k = \frac{4E_R E_r R^2 r^2 (R-r)(\alpha_r - \alpha_R)\Delta T}{(E_R R^4 + E_r r^4)(E_R R^2 + E_r r^2) + 4E_R E_r R^2 r^2 (R-r)^2} \tag{3}$$

When the fiber section can be approximately regarded as an elliptical section, the force balance and moment balance equations of composites are replaced:

$$\begin{aligned} & E_R\int_0^R\int_0^{2\pi}[\rho\sin\theta - h - \frac{1}{k}(1+\alpha_R\Delta T)]\rho \mathrm{d}\theta \mathrm{d}\rho + \\ & E_r\int_{-b}^{b}\int_{-a\sqrt{1-y^2/b^2}}^{a\sqrt{1-y^2/b^2}}[\rho\sin\theta - h + R - b - \frac{1}{k}(1+\alpha_r\Delta T)]\rho \mathrm{d}\theta \mathrm{d}\rho = 0 \end{aligned} \tag{4}$$

$$\begin{aligned} & E_R\int_0^R\int_0^{2\pi}[\rho\sin\theta - h - \frac{1}{k}(1+\alpha_R\Delta T)]\rho^2 \sin\theta \mathrm{d}\theta \mathrm{d}\rho + \\ & E_r\int_{-b}^{b}\int_{-a\sqrt{1-y^2/b^2}}^{a\sqrt{1-y^2/b^2}}[\rho\sin\theta - h + R - b - \frac{1}{k}(1+\alpha_r\Delta T)](\rho\sin\theta + R - b)\rho \mathrm{d}\theta \mathrm{d}\rho = 0 \end{aligned} \tag{5}$$

where *a* and *b* are semimajor axis and semiminor axis of the fiber elliptical section,

respectively.

Using the above two equations to eliminate h, and the equation can be obtained:

$$k = \frac{4E_R E_r S_1 S_2 (\sqrt{S_2} - \sqrt{S_1}\sqrt[4]{1-e^2})(\alpha_r - \alpha_R)\Delta T}{(E_R S_2^2 + E_r S_1^2 \sqrt{1-e^2})(E_R S_2 + E_r S_1) + 4E_R E_r S_1 S_2 (\sqrt{S_2} - \sqrt{S_1}\sqrt[4]{1-e^2})^2} \tag{6}$$

where S_1 and S_2 are cross sectional areas of fiber and liquid crystal, and e is the eccentricity of the fiber elliptical section.

Supplementary Figures

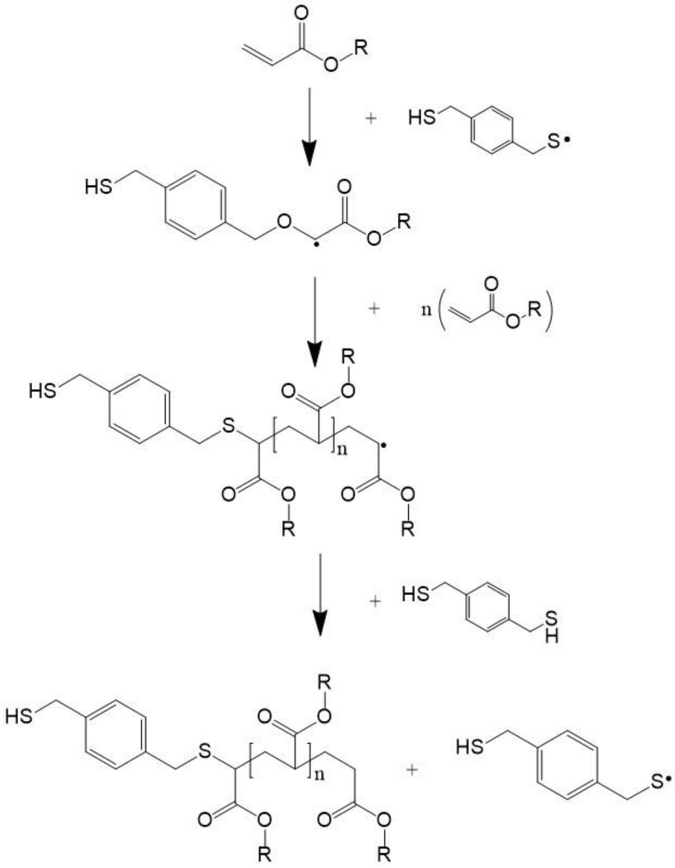

Supplementary Fig. 1 Reaction mechanism of liquid crystal components. The crosslinker BDMT used in this paper can expand the chain of liquid crystal monomers (R6M and RM257) to form liquid crystal polymers, and this figure shows the LCE polymerization principle [1].

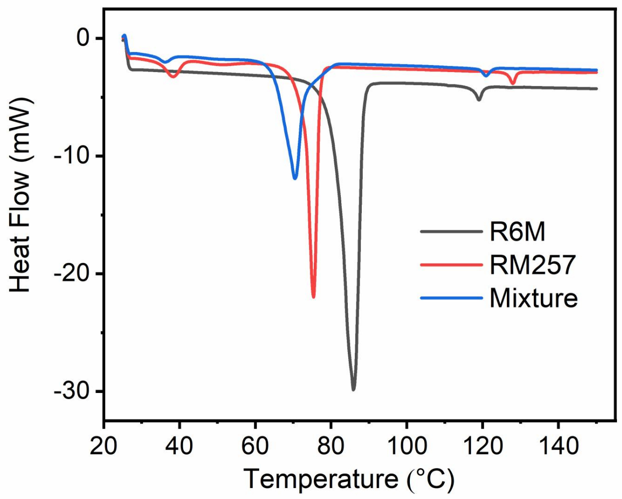

Supplementary Fig. 2 DSC test results of liquid crystal materials. The clear point of liquid crystal material was tested by DSC. The black and red curves represent the heat flow changes of R6M and RM257 respectively, while the test material used in the blue curve is a mixture with the same composition as the 4D printing material. It can be seen that the clear point of several materials is between 120 and 130 °C, so the heating temperature in the chamber should be higher than 130 °C.

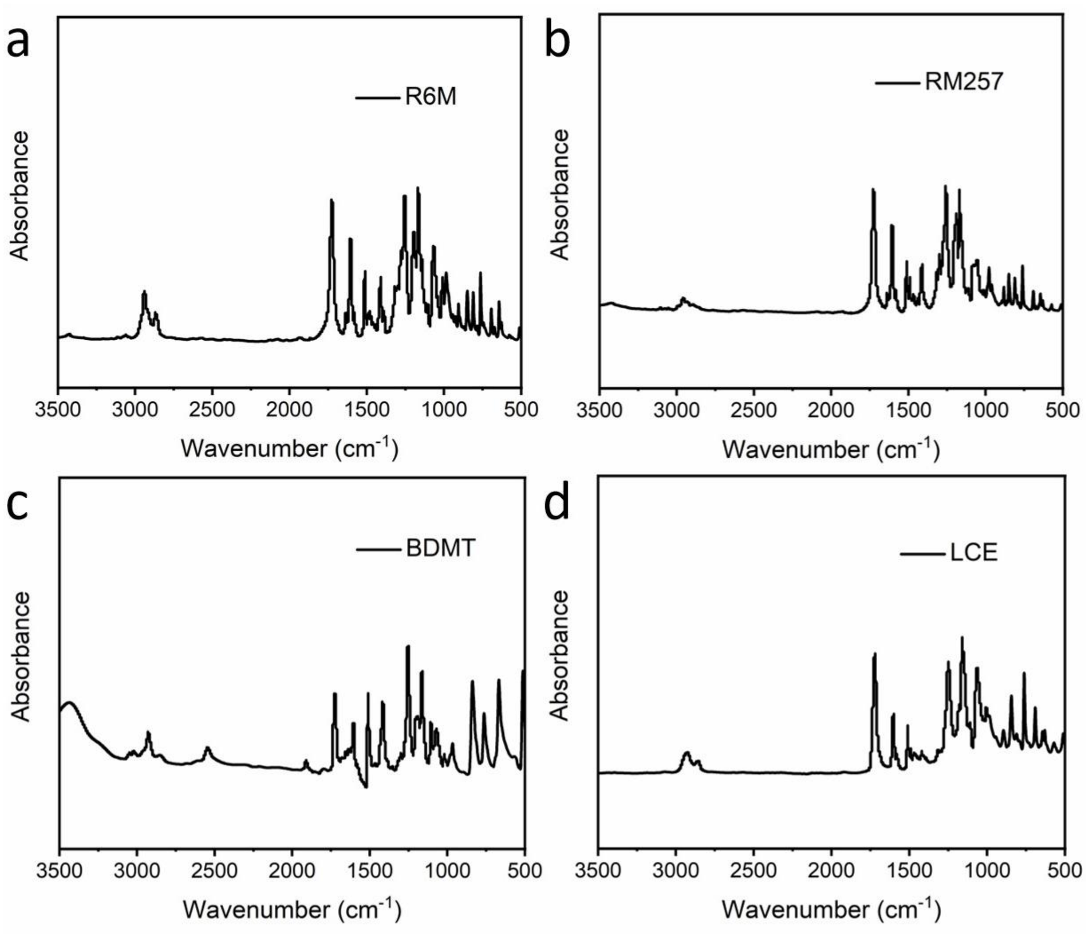

Supplementary Fig. 3 FTIR curves of liquid crystal formulas and LCE material. **a** RM257. **b** R6M. **c** BDMT. **d** LCE.

In order to determine whether the chain extension and crosslinking reactions of liquid crystal monomers are sufficient, the ink formula (including RM257, R6M, and BDMT) and the liquid crystal elastomer were characterized by infrared spectroscopy. The liquid crystal monomers RM257 and R6M both exhibit absorption peaks at 1414 cm^{-1}, which are characteristic peaks of terminal carbon carbon double bonds; The chain extender BDMT exhibits an absorption peak at 2544 cm^{-1}, which is a characteristic peak of thiol groups. As a comparison, the characteristic peaks of carbon carbon double bond and thiol group at 1414 cm^{-1} and 2544 cm^{-1} in the infrared spectrum of liquid crystal elastomer samples disappeared, indicating that the crosslinking reaction based on thioene click chemistry in the system has been basically completed.

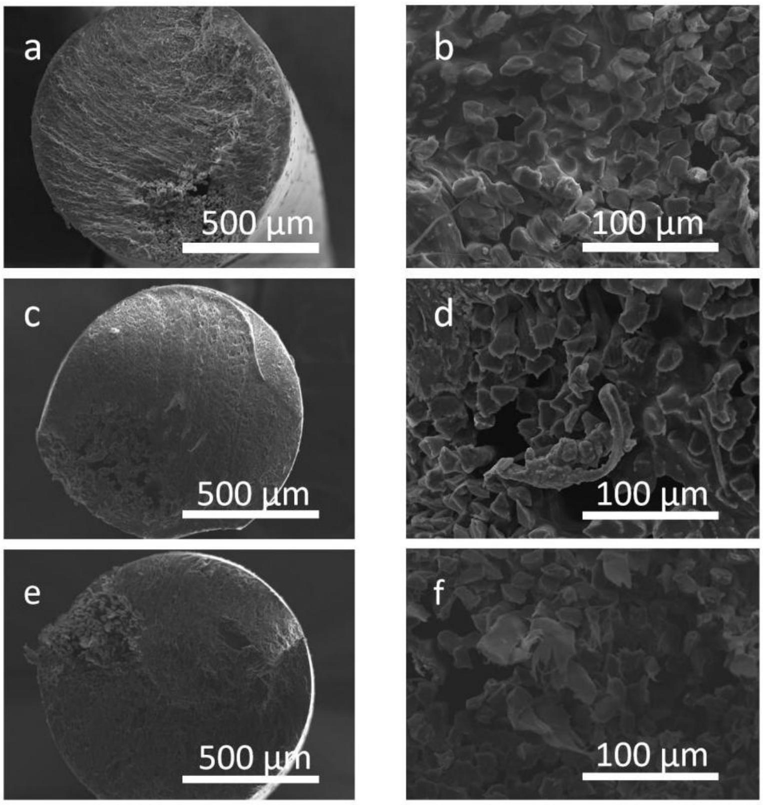

Supplementary Fig. 4 SEM images of the 4D printed composite filaments with different extrusion speeds. a, b extrusion speed of 20 mm^3 s^{-1}. **c, d** extrusion speed of 40 mm^3 s^{-1}. **e, f** extrusion speed of 80 mm^3 s^{-1}.

SEM images of cross sections of CFRLCE composite materials with a 90° inclination angle and different extrusion speeds were observed. It can be seen that the fiber bundles are not subjected to significant lateral forces during stretching, so their cross sections are close to circular, and there are holes in the fiber bundles. By comparing with Fig. 1e, it can be found that a strong off-center distribution helps to reduce the holes in the fiber and improve the interfacial properties of the two materials.

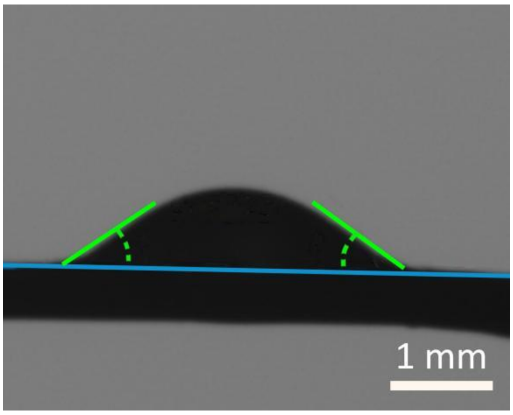

Supplementary Fig. 5 The wetting angle of the liquid crystals and continuous aramid fibers. It is necessary to prevents the liquid crystal from accumulating droplets on the surface of the fiber bundle to obtain the filament with the uniform shape during the curing process. The average wetting angle of the liquid crystals and continuous aramid fibers is 32.1° at room temperature, indicating that liquid crystals have the ability to spread on the surface of the fiber bundle.

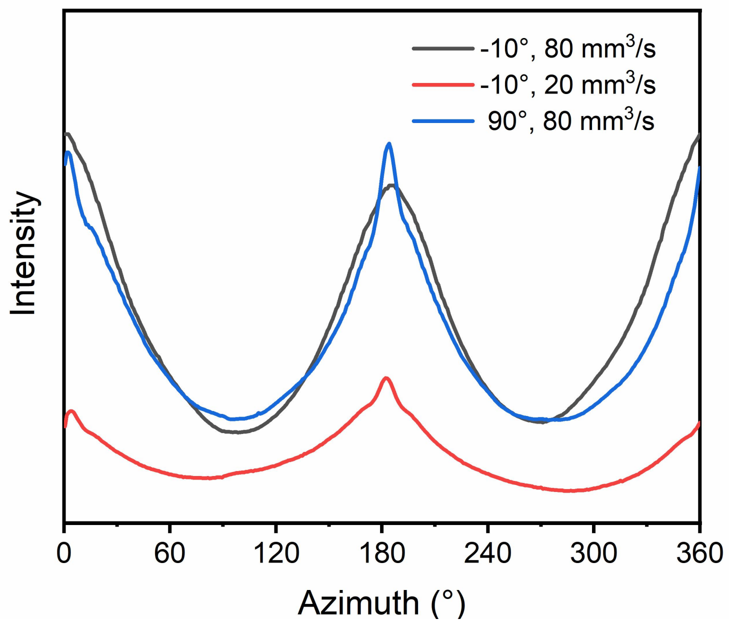

Supplementary Fig. 6 Azimuthal plots for the XRD results of the LCE materials. The XRD tests of LCEs with different extrusion speeds and tensile inclination angles were carried out, indicating that extrusion speed has a significant impact on the orientation of liquid crystal materials.

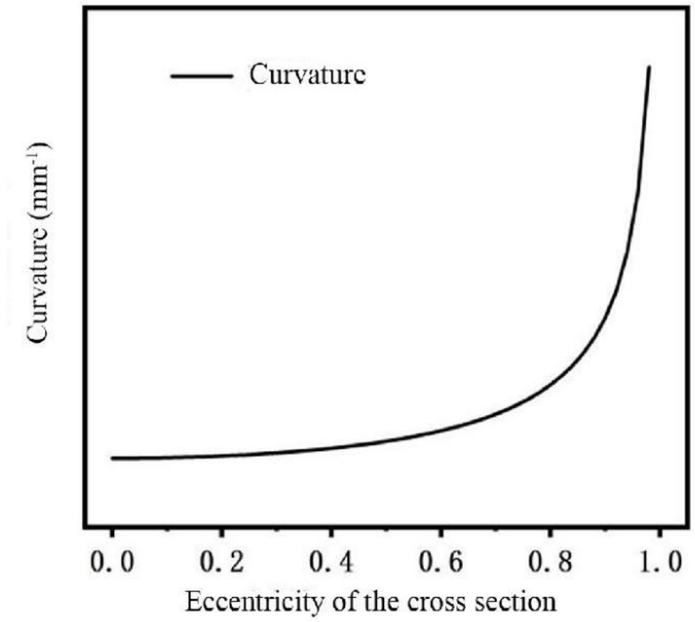

Supplementary Fig. 7 The relationship between the deformation curvature and aspect ratio of the fiber section shape. The theoretical analysis of the influence of the cross-sectional shape of the fiber bundle on the deformation curvature (Supplementary Discussion) indicates that the cross-sectional shape with a higher elliptical eccentricity corresponds to a significantly higher thermal-induced bending deformation ability.

Supplementary Tables

Supplementary Table 1. Optimized printing parameters of CFRLCEs.

Tensile inclination angle	-10°
Extrusion rate	80 mm^3 s^{-1}
Printing temperature	30 °C
Light curing time	10 min
Diameter of the nozzle	0.8 mm

Supplementary References

1 Hebner, T. S., Fowler, H. E., Herbert, K. M., et al. Polymer Network Structure, Properties, and Formation of Liquid Crystalline Elastomers Prepared via Thiol–Acrylate Chain Transfer Reactions. *Macromolecules* **54** 11074 (2021).
