# Supplementary material for: Programmable spatial deformation by controllable off-center freestanding 4D printing of continuous fiber reinforced liquid crystal elastomer composites
Source: Nat Commun. 2023 Jun 30;14:3869. doi: 10.1038/s41467-023-39566-3 (PMC10313695; doi:10.1038/s41467-023-39566-3)
Supplement: Supplementary file 3 — Description of Additional Supplementary Files [file 41467_2023_39566_MOESM3_ESM.pdf]

File Name: Supplementary Movie 1.

Description: Deformation process.

File Name: Supplementary Movie 2.

Description: 4D printing process of an L-shaped truss (played at 3x speed).

File Name: Supplementary Movie 3.

Description: 4D printing process of a triangular truss (played at 3x speed).
